# The Reliability and Feasibility of the HESPER Web to Assess Perceived Needs in a Population Affected by a Humanitarian Emergency

**DOI:** 10.3390/ijerph18041399

**Published:** 2021-02-03

**Authors:** Karin Hugelius, Charles Nandain, Maya Semrau, Marie Holmefur

**Affiliations:** 1 Faculty of Medicine and Health, School of Health Sciences, Örebro University, 701 82 Örebro, Sweden; marie.holmefur@oru.se; 2 School of Education and Social Sciences, International Leadership University, Nairobi 00200, Kenya; cnandain@kenya.ilu.edu; 3 Centre for Global Health Research, Brighton and Sussex Medical School, Brighton BN1 9PX, UK; m.semrau@bsms.ac.uk

**Keywords:** humanitarian emergencies, mental health, needs assessment, refugee health, disaster health

## Abstract

Needs assessment is essential in the humanitarian response, and perceived needs can be associated with the levels of health in populations affected by humanitarian emergencies. This study aimed to evaluate the reliability and feasibility of The Humanitarian Emergency Settings Perceived Needs Web (HESPER Web) in a humanitarian context and to compare perceived needs of a random walk study sample with a self-selected study sample recruited though social media. The study context was the Dadaab refugee camp in Kenya. An alternate forms reliability evaluation and a feasibility evaluation was conducted. In total, 308 refugees participated in the study. HESPER Web was found to be reliable and usable for assessing needs, with an intraclass correlation coefficient (ICC) of 0.88, Cohen’s κ between 0.43 and 1.0 and a first priority need rating match of 81%. The HESPER Web was positively experienced, and the self-recruited study sample reported similar levels of needs and similar demographics as the randomized sample. The participants reported several unmet needs. HESPER Web offers a reliable tool for needs assessment in humanitarian emergencies where web-based surveys are considered as practical and suitable. It offers new possibilities for conducting remote assessments and research studies that include humanitarian populations that are rarely included in such evaluations.

## 1. Introduction

Humanitarian health has been suggested as a key research priority and an essential part of global health initiatives in emergencies [1]. In 2020, nearly 168 million people worldwide are estimated to be in need of assistance or protection due to humanitarian emergencies, such as conflicts or natural disasters [2]. Needs assessment is one of the fundamental cores of the humanitarian health response, both in long-lasting humanitarian settings and after sudden-onset disasters. A proper and well-designed needs assessment lays the foundation for a coherent, efficient and trustworthy humanitarian response to any emergency [3]. Many of the humanitarian emergencies are long-lasting crises, and the majority of all people considered as affected by humanitarian emergencies live in designated areas, such as camps [3]. Health and well-being among people living in such camps include a wide range of potential health problems: infectious diseases, chronic conditions, injuries, malnutrition, gender-based violence, mental health problems and disruption of cultural and social conventions [4]. The daily life of affected people living in camps is fraught with unmet basic needs [5]. Having a higher level of perceived needs has been found to predict a greater level of psychological distress [6]. Therefore, a reliable assessment of perceived needs can be said to be the fundament in order to understand mental health and other health problems among people in vulnerable situations [7].

The development and availability of scientifically and contextual feasible instruments to assess health and needs in humanitarian emergencies is strongly needed [1]. The Humanitarian Emergency Settings Perceived Needs (HESPER) scale was developed to provide a quick and reliable way to assess the perceived needs of affected people in humanitarian emergencies, including complex emergencies, conflicts and natural disasters [8]. The HESPER scale was developed by the World Health Organization and Institute of Psychiatry at King’s College London, based on literature studies, experts on humanitarian assessments and several pilot- and field tests including different samples of populations affected by different kinds of humanitarian emergencies. A detailed description of the development and testing of the scale has been reported elsewhere [8]. The original HESPER was designed to gather data through individual face-to-face interviews and paper surveys. Today, an increasing part of the world population has access to Internet connections. About 93% of all displaced people in the world have access to a mobile network, and many have access to the Internet, through a mobile connection, broadband in schools, community Internet cafés or other sources. Additionally, in rural areas, the coverage and quality are progressively improving [9]. Internet-based data gathering offers quicker data collection and analysis and fewer internal dropouts and processing errors, and is often a more economical alternative to other types of surveys [10]. To combine the strengths of Internet-based data collections and a scale measuring perceived needs among humanitarian populations, we developed HESPER Web, a self-administrated web-based version of the original HESPER [11]. The HESPER Web can be administrated through a web link and answered on a computer, tablet or mobile phone and the first psychometric evaluation of HESPER Web showed very good reliability and feasibility among a study sample of asylum seekers in Sweden [11]. In order to further evaluate the HESPER Web, a field test in a large scale humanitarian context was necessary.

This study had three aims; (1) to evaluate the reliability and feasibility of HESPER Web in a large-scale humanitarian context, (2) to compare the demographics and means of the perceived needs of a random walk method study sample and a convenient, self-selected study sample recruited though social media and (3) to describe the perceived needs within the study sample.

## 2. Materials and Methods

The study consisted of two parts: an alternate forms reliability evaluation and a feasibility evaluation. When analyzing the reported needs, data from both the alternate forms evaluation and the feasibility evaluation were used.

### 2.1. Study Setting

The Dadaab refugee camp in eastern Kenya has been operative for about 26 years and consists of three smaller camps, Dagahaley, Ifo and Hagadera. In February 2019, the camps hosted about 205,000 refugees [6]. The absolute majority of the Dadaab population are refugees from Somalia, and about 50% of all people living in Dadaab are male. The United Nations High Commissioner for Refugees (UNHCR) is the operational manager of the camps, and all services, such as housing, food, water supply, basic health care and schools, are free for registered refugees [12]. The Hagadera refugee camp houses about 83,940 people, where 50% are male. Hagadera has 10 schools. In a household survey conducted in 2017, 43% of all households reported English as their first language [13]. The Internet is available through a 3G connection (via mobile networks) or by broadband in the 10 schools, one adult literacy centre and one ICT training centre [13]. The study context for this study was the Hagadera refugee camp and the data collection was conducted in February 2019.

### 2.2. Instrumentation

The HESPER scale consists of 26 fixed questions covering physical, psychological and social needs [8]. The ratings are made by interviewers in a face-to-face interview with affected persons by asking whether a certain need is perceived as a “serious problem” or not. In addition, the affected person can add other needs if not covered by the original 26 stated needs. After reporting their needs, the affected person is asked to prioritize the three most serious perceived needs. A total sum score can be calculated by adding up the total number of “serious problem” ratings [8].

The HESPER Web is a newly developed web based, self-administrated survey version of the original HESPER scale [11]. The HEPSER Web could be accessed by a web link by a mobile phone, tablet or computer. In addition to the 26 questions regarding certain needs and the prioritizing question in the HESPER Web, study specific feasibility questions were added to the HESPER web survey. These questions were: how long did it take for you to fill in the survey? were the questions easy to understand? what mean did you use for answering the survey? did you experience any technical problems when answering the survey? did you suffer from any harm by filling in the survey? could you answer the survey in privacy? and how did you get the invitation for this survey?

### 2.3. Alternate Forms Evaluation

The alternate forms reliability between the original HESPER scale and HESPER Web was evaluated using a voluntary convenience study sample of 50 study participants from the camp. Based on a power analysis that indicated a need for a minimum of 19 participants in both data collections in order to detect a statistically significant correlation and a power of 90%, and previous experiences from conducting alternative forms evaluation [11,14,15], a sample size of 50 was chosen. Inclusion criteria were that the person should be at least 18 years old, have access to the Internet by mobile phone, tablet or computer and be able to participate in the interview using the English language. For all participants, the HESPER interview was made prior to the web survey, due to practical reasons. The HESPER interviews were conducted by two male and two female volunteer assistants trained during a six-hour training session in accordance with the HESPER manual. Using a cluster random sampling method, four square areas within the Hagadera camp were first selected by lot to be included in the study [13]. Thereafter, the households asked to participate were selected using a kind of random walk method [8], where every second house in a direction pointed at by spinning a pencil was visited. In the first household, the first person to approach the interviewers was asked to participate. In the second household visited within the cluster, the second person seen by the interviewers was asked to participate, and so on. If any of the persons selected could not participate for any reason, the next person in the household was asked. The interviewers estimated that in every third household, there was no person eligible for participation. If so, the interviewers continued to the next household. A code list was used to group the HESPER scale and HESPER Web answers. Both data collections were answered anonymously, using the specific code only as reference in the web survey. The participants got a personally written reminder note from the interviewer, with the code and the link to HESPER Web, asking them to complete it within 48 h. The time between the HESPER interview and that taken to answer HESPER Web varied from a few hours up to three days.

### 2.4. Feasibility Evaluation

**Figure 1:**
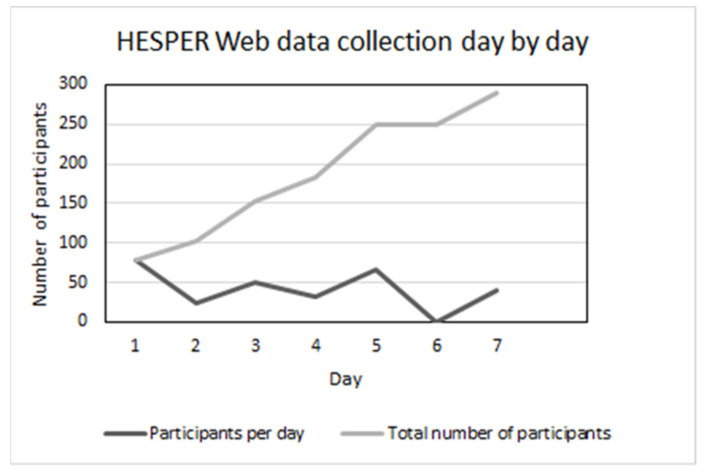
Number of participants per day in the HESPER Web voluntary, self-recruited study sample.

The sample for the feasibility evaluation of HESPER Web was conducted with 289 voluntary study participants who were recruited by advertising the study in the adult training centre, secondary schools and internet- and communication centers in Hagadera. Additionally, digital advertising on Facebook and on three specific pages aimed at people living in Hagadera or other Dadaab camps was used. Inclusion criteria were that the study participant should be at least 18 years old, have access to the Internet by mobile phone, tablet or computer and be able to participate in the survey using the English language. The data collection period lasted for seven days (see Figure 1). The web survey was anonymous, and there were no limitations on answers from the same IP address, in order to allow several responders to use the same computer, tablet or mobile phone to answer the survey. Data were saved in a secured research database at Orebro University in Sweden.

### 2.5. Analysis

For the alternate forms reliability between the HESPER scale and HESPER Web, intraclass correlation coefficients (ICCs), two-way mixed and absolute agreement [16], of the total number of reported serious needs was calculated. To assess agreement on an item level and the percentage match between first priority needs in the HESPER scale and HESPER Web, Cohen’s κ was used. Additionally, descriptive statistics for analyzing the feasibility questions and the reported needs were used. SPSS software (IBM Corp. Released 2016. IBM SPSS Statistics for Windows, Version 24.0. Armonk, NY: IBM Corp) was used to conduct the statistical analysis. A significance level of *p* ≤ 0.05 was used.

### 2.6. Ethical Considerations

Informed consent was obtained by each study’s participants before participating in the interview and/or web survey. The study was approved by the Regional Ethical Committee in Sweden (ID 2017/481) and the National Commission for Science, Technology and Innovation (NACOST) in Kenya. Permission to develop and evaluate the HESPER Web was obtained from the WHO.

## 3. Results

In total, 308 individuals participated in the study: 50 in the alternate forms evaluation and 289 in the feasibility evaluation. Table 1 shows the demographics. There was no significant difference between the HESPER interview sample (*n* = 50) and the HESPER Web feasibility evaluation sample (*n* = 289) regarding gender (Chi 2 test, *p* = 0.33), age (Chi 2 test, *p* = 0.78) or present location (Chi 2 test, *p* = 0.99) but for country of origin (Chi 2 test, *p* < 0.001). There was no significant difference between the participants who participated in both the HESPER interview and the web survey, and those who dropped out from the survey (gender (Chi 2 test, *p* = 0.61), age (Chi 2 test, *p* = 0.50) or present location (Chi 2 test, *p* = 0.99) and country of origin (Chi 2 test, *p* = 0.97).

### 3.1. Alternate Forms Evaluation

Of the 50 participants recruited for the alternate forms reliability evaluation and who participated in the HESPER interview, there were 19 dropouts who did not answer the HESPER Web. The alternate form results were therefore based on 31 participants.

The ICC was 0.88 (CI 0.60–0.91) between the HESPER scale and HESPER Web. For the item-by-item evaluation between the HESPER scale and HESPER Web, Cohen’s κ was calculated, and it varied between 0.43 (for the item concerning safety) and 1.0 (for the item relating to law and justice in the community and other serious problems), see Table 2. Regarding the first priority need rating, an overall match of 81% was found between the HESPER scale and HESPER Web.

### 3.2. Feasibility Evaluation

Answering the HESPER Web survey was quicker than being interviewed for many of the study participants (*p* < 0.001, see Table 3). The questions asked in HESPER Web were considered to be easy to understand, and no participant reported experiencing harm caused by the survey. About 86% of all study participants could answer HESPER Web in privacy (see Table 4). An absolute majority of the participants used their own mobile phones to answer the survey (60%), followed by a significant number who used someone else’s computer or tablet, including the school’s or ICT center’s (19%). About 13% used someone else’s mobile phone or their own computers or tablets (4%).

### 3.3. Differences in Demographics between the Randomized Study Sample and Self-Selected Sample

No significant difference in the total reported numbers of needs could be observed between the HESPER scale and HESPER Web study samples (*p* = 0.067, paired *t*-test) or when comparing HESPER Web (alternate forms) and HESPER Web (feasibility evaluation; two-sample *t*-test, *p* = 0.132). No significant difference in gender (Chi 2 test, *p* = 0.670) or age (two-sample *t*-test, *p* = 0.810) between the HESPER interview sample and the HESPER Web self-selected sample was observed.

### 3.4. Perceived Needs

When reporting results on their perceived needs, a total sample of 320 people was used, including all study participants who answered HESPER Web (as part of the feasibility evaluation (*n* = 289) or the alternate forms evaluation (*n* = 31)), and not the ones who only participated in the HESPER interviews. When reporting results on their perceived needs, a total sample of 320 people was used, including all study participants who answered HESPER Web (as part of the feasibility evaluation (*n* = 289) or the alternate forms evaluation (*n* = 31)), and not the ones who only participated in the HESPER interviews.

The mean number of reported needs among the study participants in the HESPER scale was 4.52 (SD 3.2, range 1–15). The frequency of reported needs in total and sorted on gender is shown in Table 5.

There was no significant difference between males and females regarding the mean of the total number of reported needs (Student’s *t*-test: male mean 5.88, SD 3.9, (95% CI: 5.27; 6.48), range 0–21), female mean 6.43, SD 3.9, (95% CI: 5.50;7.0), range 1–19, *p* = 0.765), but there were some differences in what kind of needs were reported (see Table 5)

## 4. Discussion

HESPER Web was found to be reliable and usable for assessing perceived needs among refugees living in a large-scale humanitarian context such as the Dadaab refugee camp. The use of a web-based survey was positively experienced by the study participants, and the voluntary self-recruited study sample reported similar levels of needs and similar demographics regarding gender and age to the walking methods randomized study sample. The participants reported several unmet needs, and there were some differences in the kinds of needs identified depending on gender.

The alternate forms evaluation showed overall good correspondence between the HESPER scale and HESPER Web in general (ICC 0.88) and on an item by item level (Cohen’s κ from 0.43 to 1.0) [17]. The item with the lowest consistency was the question on perceived problems caused by security issues. The reason for this might be that the current level of security varied a lot from day to day and from location to location within the Dadaab camp. Additionally, there was an observed difference when reporting on educational needs for children. However, this item was frequently reported in the larger sample (Table 5) and therefore, we could not explain the difference noted in the comparison between the HESPER and the HESPER Web. The association for the first priority rating was very good (81%) [17], showing that HESPER Web reliably can be used to assess the most serious perceived needs instead of or as a complementing data collection method to the HESPER interview. However, it should be noted that the timeframe between first and second data collection was short (from a few hours up to 3 days), and that might have influenced the results. It would have been preferred with a longer timeframe between the two measurements, but due to security regulations, repeated visits could not be conducted. The short timeframe may have resulted in that participants remembered their answers from the first data collection, which may have contributed to a slightly overestimated alternate forms reliability coefficient. Even when taking this into account the alternate forms reliability between the two forms of administration of the HESPER is good.

In the HESPER manual, strategies to perform data collection in order to ensure a proper study sample are described. When using web-based methods, the same procedures may be used with the difference that the study participant answers the web-based survey instead of taking part in an interview. If advertising the survey on social media or physical locations, the study sample will be a convenience sample. This study suggests that the study samples from the walk-around sampling method and the self-selected sample were similar, regarding both their demographics and the mean number of reported needs. However, it should be noted that the number of study participants differed between the samples, and the exact number of study participants needed for generalization of a web based, not randomized data collection cannot be concluded from this study.

When conducting HESPER interviews face to face, the interviewer could interact with the person and, if needed, provide specific advice or refer to, for example, psychosocial support. When using a self-administrated web-based survey, this is no longer an option. Therefore, it is of extra importance for a survey provider to state the limitations and to provide practical support and to state where the study participants should turn for help in case of an immediate need for such support. In addition, a web-based survey may offer new possibilities to direct people who report need of support, and guide them on where to turn for available support.

HESPER Web has shown potential in reducing several challenges that are common in disaster or humanitarian emergency health research related to the practical possibilities of physically reaching or visiting an area, security concerns and ethical considerations, such as the possibility of being anonymous [18,19,20]. HESPER Web can offer possibilities for conducting assessments and research studies that include populations that are rarely included in such evaluations, such as people who constantly move around, people evacuated from the study area or those who do not have access to a fixed address [20]. In addition, the tool may be used for longitudinal studies on perceived needs [11]. However, not all study populations or contexts are suitable for web-based needs assessment or research. The reasons may be several, including limited access to the Internet or a means for answering the survey, limited privacy when answering the survey or illiterate or severely traumatized populations where personal contact may be necessary to assess mental health or provide support. The responsibility of using a valid and proper instrument and data collection procedure and considering the context and study population is always the researcher’s or the head of the organization’s responsibility, and not that of the affected population.

In this survey, the study participants reported several needs, although, they were settled in a long-lasting state of displaceability. Web-based methods for assessing mental health have been suggested to provide a better picture of the actual situation while offering anonymity and reducing stigma in the interview situation [19]. Higher levels of perceived needs can significantly predict psychological distress and lower levels of functioning [6]. It has been suggested that further emphasis should be put on developing tools for community mental health providers to enhance reach and effects from mental health interventions in low- and middle-income populations [21]. To assess perceived needs and plan for mental health interventions also in populations with long lasting displaceability seems therefore reasonable. Additionally, it has been suggested to further explore the use of self-help digital mobile applications used in community based mental health interventions in for example refugee camps [21]. For such purposes, the HESPER Web could be a feasible tool, but need to be further evaluated.

This study had several limitations. It would have been preferable to let half of the study participants in the alternate form evaluation answer the HESPER scale first, and then HESPER Web, and the other half in the opposite order. Due to security reasons, that could not be done. Additionally, such a strategy was however considered to increase the risk of dropouts between the two data collections and therefore dismissed. The use of “random walk sampling” is usually not the preferably choice of the sampling method for research studies. However, it was considered as the best possible option, given the security environment and practical possibilities. The way the “random walk sampling” was used in this study can be described as a combination of a “spin the pen” sampling and a clustered sampling method and is recommended for research in humanitarian emergencies when other, traditional methods are not possible or suitable [22].

When conducting research in humanitarian emergency settings, the research needs to be done with, and for populations affected in order to determine interventions that are feasible and appropriate for the context [1]. In this study, several actors with extensive knowledge and involvement in local processes were involved in planning, practical data collection and the analysis of this study, including local UN agencies, NGOs and academic partners. Partnerships with local individuals ensure a local perspective and add value to the interpretations of the results [23,24]. However, the study participants themselves were not actively engaged in parts other than the data collection. The active engagement of the people affected is essential to ensure that the response is based on their actual needs and supports their recovery [23]. Asking the refugees themselves for their perceived needs may, therefore, contribute to both community engagement and individual recovery [25]. However, little is known about refugee participation in the development of policies and programs that matter to their health and well-being. Such participation is fundamental for more sustainable and responsive projects [4], and a plan for the dissemination of the results should, therefore, be considered in future projects.

## 5. Conclusions

HESPER Web was found to be reliable and usable for assessing perceived needs among a population affected by a humanitarian emergency. The use of a web-based survey was positively experienced by the study participants, and the voluntary, self-recruited study sample reported similar levels of needs and similar demographics regarding gender and age to the randomized study sample. HESPER Web offers a reliable and feasible tool for assessment of needs in situations where web-based surveys are considered as practical and suitable. It offers new possibilities for conducting remote assessments and research studies that include humanitarian populations that are rarely included in such evaluations.

## Figures and Tables

**Table 1:** Demographics of the study participants.

		Alternate Forms Reliability Evaluation		Feasibility Evaluation
		HESPER (Interviews)	HESPER Web (Web Survey)	HESPER Web (Web Survey)
*N*		50	31	289
	No participation *n*	-	19	-
Gender, *n* (%)	Male	28 (56)	18 (58)	152 (53)
	Female	22 (44)	13 (42)	136 (47)
	Missing	0	0	1
Age	Mean (SD)	28 (7.2)	31 (6,8)	28 (8,0)
	Min	19	19	18
	Max	45	44	54
	Missing	0	1	4
Country of	Somalia	41 (82)	28 (90)	234 (81)
Origin, *n* (%)	Sudan	1 (2)	0	23 (8)
	Stateless	0	0	4 (1)
	Other	6 (12)	0	19 (6)
	Missing	2	3	9
Location,	Hagadera camp	50 (100)	31 (100)	244 (85)
*n* (%)	Dadaab, other			10 (3)
	Kakuma camp			28 (10)
	Other			3 (1)
	Missing	0	1	4
Mean of needs (SD) Range of needs		4.7 (3.3) 1–15	5.1 (3.0) 1–15	5.0 (4.1) 1–19

SD = Standard Deviation.

**Table 2:** Persons reporting specific needs and Cohen’s κ between the HESPER and HESPER Web, per item.

Item ^1^	HESPER Interviews	HESPER Web	Cohen’s κ
	***n* (%)**	***n* (%)**	
*n*	50	31	
Drinking water	0 (0)	0 (0.0)	*n*/a
Food	4 (8)	2 (4)	0.70
Place to live in	8 (16)	6 (12)	0.59
Toilets	0 (0)	3 (6)	*n*/a
Keeping clean	1 (0)	2 (4)	0.659
Clothes, shoes, bedding or blankets	11 (22)	5 (10)	0.60
Income or livelihood	28 (56)	22 (44)	0.77
Physical health	6 (12)	6 (12)	0.62
Health care	15 (30)	8 (16)	0.83
Distress	9 (18)	6 (12)	0.81
Safety	7 (14)	3 (6)	0.43
Education for your children	0 (0)	6 (12)	*n*/a ^2^
Care for family members	3 (6)	5 (10)	0.57
Support from others	12 (24)	8 (16)	0.56
Separation from family members	14 (28)	11 (22)	0.93
Being displaced from home	21 (42)	15 (32)	0.87
Information	7 (14)	4 (8)	0.87
The way aid is provided	8 (16)	6 (12)	0.89
Respect	10 (20)	7 (14)	0.91
Moving between places	15 (30)	12 (24)	0.93
Too much free time	15 (30)	8 (16)	0.82
Law and justice in your community	7 (14)	3 (6)	1.00
Safety or protection from violence for women in your community	3 (6)	*n*/a	*n*/a ^2^
Alcohol or drug use in your community	1 (2)	*n*/a	*n*/a ^2^
Mental illness in your community	0 (0)	*n*/a	*n*/a ^2^
Care for people in your community who are on their own	2 (6.5)	*n*/a	*n*/a ^2^
Other serious problems	1 (2)	1 (2)	1.00

^1^ Items presented in the HESPER Web order; ^2^ Kappa value could not be calculated due to zero answers in one or more samples.

**Table 3:** Time to answer the survey.

	HESPER (Interview)	HESPER Web
*n*	50	302
<10 min	23	234
11 to 20 min	13	22
>20 min	2	0
Missing	4	54

Chi 2 test, *p* = 0.00, Cramer’s V = 0.503.

**Table 4:** Feasibility evaluation questions for HESPER Web.

Total HESPER Web Answers *N* = 289	Yes *n* (%)	No *n* (%)	Don’t Know *n* (%)	Missing Data *n* (%)
Questions were easy to understand	257 (89)	7 (2)	2 (0)	23 (8)
Experienced technical problems	36 (12)	237 (82)	6 (2)	10 (6)
Experienced harm from filling out the survey	0 (0)	267 (93)	5 (2)	17 (6)
Possible to answer the survey in private	247 (86)	10 (4)	6 (2)	26 (9)

**Table 5:** Reported serious needs, item by item.

Item	Total Persons Reporting the Need *n* (%)	Male Reporting the Need *n* (%)	Female Reporting the Need *n* (%)	Differences between Gender *p*-Value ^a^
*N*	320	168	152	
Drinking water	0 (0)	0 (0)	0 (0)	-
Food	11 (3)	6 (4)	5 (3)	0.539 ^b^
Place to live in	60 (19)	30 (18)	30 (20)	0.345
Toilets	3 (1)	2 ()	1 (0)	0.539 ^b^
Keeping clean	27 (0)	9 (5)	18 (12)	0.023
Clothes, shoes, bedding or blankets	60 (19)	29 (17)	31 (20)	0.363
Income or livelihood	160 (50)	88 (52)	72 (47)	0.332
Physical health	38 (12)	15 (9)	23 (15)	0.087
Health care	98 (31)	45 (27)	53 (35)	0.077
Distress	78 (24)	32 (19)	46 (30)	0.017
Safety	55 (17)	18 (11)	37 (24)	0.001
Education for your children	18 (6)	8 (5)	10 (7)	0,346 ^b^
Care for family members	8 (3)	5 (3)	3 (2)	0.380 ^b^
Support from others	59 (18)	19 (11)	40 (26)	0.001
Separation from family members	96 (30)	52 (31)	44 (30)	0.400
Being displaced from home	87 (27)	34 (20)	53 (35)	0.003
Information	51 (16)	28 (17)	23 (15)	0.397
The way aid is provided	91 (28)	51 (30)	40 (26)	0.308
Respect	65 (20)	44 (16)	21 (14)	0.006 ^b^
Moving between places	43 (13)	33 (20)	10 (7)	0.001 ^b^
Too much free time	111 (35)	72 (43)	39 (26)	0.001
Law and justice in your community	105 (33)	66 (39)	39 (26)	0.015
Safety or protection from violence for women in your community	51 (16)	26 (15)	25 (16)	0.076
Alcohol or drug use in your community	16 (5)	11 (7)	5 (3)	0.093 ^b^
Mental illness in your community	1 (0)	1 (0)	0 (0.0)	0.534 ^b^
Care for people in your community who are on their own	4 (1)	2 (1)	2 (3)	0.668 ^b^
Other	5 (2)	1 (0.0)	4 (3)	0.147
Mean of total needs (SD)	6.14	5.88	6.43	0.765 ^c^

Bolded number indicate a significant difference (*p* ≤ 0.05). a = calculated with the Chi^2^ test or if indicated with ^b^ where the Fischer’s exact test was used, or ^c^ where the Student’s *t*-test was used.

## Data Availability

The datasets analyzed during the current study are not publicly available due to the Swedish law on ethical approval for research but are available from the corresponding author on reasonable request.
